# Light-Controlled Modulation and Analysis of Neuronal Functions

**DOI:** 10.3390/ijms232112921

**Published:** 2022-10-26

**Authors:** Carlo Matera, Piotr Bregestovski

**Affiliations:** 1 Department of Pharmaceutical Sciences, University of Milan, 20133 Milan, Italy; 2 Institut National de la Santé et de la Recherche Médicale, Institut de Neurosciences des Systèmes, Aix-Marseille University, 13005 Marseille, France; 3 Institute of Neurosciences, Kazan State Medical University, 420111 Kazan, Russia; 4 Department of Normal Physiology, Kazan State Medical University, 420111 Kazan, Russia

Light is an extraordinary tool allowing us to read out and control neuronal functions thanks to its unique properties: it has a great degree of bioorthogonality and is minimally invasive; it can be precisely delivered with high spatial and temporal precision; and it can be used simultaneously or consequently at multiple wavelengths and locations. In the last 15 years, light-based methods have revolutionized the way we analyse and control biological systems. It turns out that with the help of light it is possible to study the functions of cells and cell ensembles [1,2,3], regulate the functions of deoxyribonucleic acid (DNA) [4], perform the photocontrol of peptide conformations [5], modulate the activity of voltage-gated and receptor-operated channels [6,7,8], and measure the concentrations of ions [9,10] and other cellular components [11,12]. It has become possible to control the behaviour of organisms [13,14], as well as to explore new ways of treating certain diseases [15,16,17]. Three novel approaches have been emerging more than others: optogenetics, optosensorics, and photopharmacology (optopharmacology).

Optogenetics is an elegant method that combines optical technology and genetic engineering to control the functions of biological systems (cells, tissues, organs, organisms) genetically modified to express photosensitive proteins [2]. By stimulating with light, this method provides high-spatiotemporal and high-specificity resolutions, in contrast to conventional pharmacological or electrical stimulation [18,19]. Optogenetics provides a route to study synaptic circuits [20] and underlying movement diseases [21,22] and has become an effective technology to revolutionize brain research for the therapy of vision [23,24,25], cardiovascular [26], and neurodegenerative disorders [27,28]. It has also been extended to other biomedical fields [29,30].

Optosensorics is the direction of research exploring the possibility of non-invasively monitoring intracellular ion concentrations and the activity of enzymes, lipids, and other cellular components using specific optical sensors. Most of the sensors developed in the past years are genetically encoded tools. These probes possess fluorophore groups capable of changing fluorescence when interacting with certain ions or molecules [31]. The use of modern optical and fluorescent technologies in combination with molecular genetic techniques has provided the possibility for the biosensoric monitoring of reactive oxidative species [32,33], cAMP [34], glucose [35,36], glutamate [37,38], pyruvate [39], lactate [40], different ions [41,42], and neuronal activity [43].

In contrast to the above-mentioned methods, photopharmacology relies on synthetic photoswitchable ligands that are externally supplied and usually does not require genetic manipulation. Photopharmacological agents are obtained via the incorporation of a molecular photoswitch (e.g., azobenzene and its derivatives) into the structure of a biologically active compound, so that light can be used as an external signal to “switch” the ligand conformation and hence its activity between two different states (ideally “off” and “on”) [44,45]. This is ultimately performed to achieve control over biological systems with a high degree of temporal and spatial precision for investigational and therapeutic purposes. Potential applications of photopharmacology include neurobiology [46,47,48], cancer [49,50], microbial infections [51], pain [52,53], and blindness [54,55], among others.

In this Special Issue, several papers focus on neurobiological aspects, using light as a tool for experimental analysis. In the paper published by Gerasimov et al., the authors describe an optogenetic approach to stimulate astrocytes with the aim to modulate neuronal activity [56]. They applied light stimulation to astrocytes expressing a version of ChR2 (ionotropic opsin) or Opto-α1AR (metabotropic opsin). Optimal optogenetic stimulation parameters were determined using patch-clamp recordings of hippocampal pyramidal neurons’ spontaneous activity in brain slices as a readout. They observed that the activation of the astrocytic Opto-α1AR, but not ChR2, results in an increase in the fEPSP slope in hippocampal neurons. The authors conclude that Opto-α1AR expressed in hippocampal astrocytes provides an opportunity to modulate the long-term synaptic plasticity optogenetically and suggest that this approach may potentially be used to normalize the synaptic transmission and plasticity defects in a variety of neuropathological conditions, including models of Alzheimer’s disease and other neurodegenerative disorders.

In another work, Abd El-Aziz et al. used an optogenetic system to study the activity of the epithelial Na^+^ channel (ENaC) [57]. The activity of ENaC is strongly dependent on the membrane phospholipid phosphatidylinositol 4,5-bisphosphate (PIP2). PIP2 binds two distinct cationic clusters within the N termini of β- and γ-ENaC subunits (βN1 and γN2). The purpose of this study was to determine whether each independent PIP2–ENaC interaction site is sufficient to abolish the response of ENaC to changes in PIP2 levels. The authors had previously determined the affinities of these sites using short synthetic peptides. In this paper, they describe their role in sensitizing ENaC to changes in PIP2 levels in the cellular system. For this purpose, they compared the effects of PIP2 depletion and recovery on ENaC channel activity and intracellular Na^+^ levels [Na^+^]_i_. They tested the effects on ENaC activity with mutations to the PIP2 binding sites using the optogenetic system CIBN/CRY2-OCRL to selectively deplete PIP2. Whole cell patch-clamp measurements showed a complete lack of response to PIP2 depletion and recovery in ENaC with mutations to βN1 or γN2 or both sites compared to wild-type ENaC. These results suggest that the βN1 and γN2 sites on ENaC are each necessary to permit maximal ENaC activity in the presence of PIP2.

Lilja et al. presented the results obtained with a novel optogenetic tool for the investigation of neuroplasticity [58]. The activation of tropomyosin receptor kinase B (TrkB), the receptor of brain-derived neurotrophic factor, plays a key role in induced juvenile-like plasticity (iPlasticity), which allows the restructuring of neural networks in adulthood. In this work, they evaluated the utility of a new, highly sensitive, optically activatable tropomyosin receptor kinase B receptor, named OptoTrkB (E281A). OptoTrkB (E281A) was successfully transduced in parvalbumin-positive (PV+) interneurons and in alpha calcium/calmodulin-dependent protein kinase type II positive (CKII+) pyramidal neurons specifically. Light stimulation through transparent skulls or even through a high-opacity barrier (intact skull and fur) promoted the phosphorylation of ERK and CREB, downstream signals of TrkB, in the neurons expressing optoTrkB (E281A) at a certain level. Their results indicate that this highly sensitive optoTrkB (E281A) receptor can be activated using wireless optogenetic methods and can thus be used for a broad range of behavioural studies. Overall, these findings show that the highly sensitive optoTrkB (E281A) can be used in iPlasticity studies of both inhibitory and excitatory neurons, with flexible stimulation protocols in behavioural studies.

The application of novel optogenetic/chemogenetic tools and advanced in vitro models, including those based on iPSC-derived cells, organoids, or utilizing 3D brain-on-chip platforms, are of great importance for the development of new therapeutic options and the assessment of aberrant neurogenesis in Parkinson’s-type neurodegeneration. In their review article, Salmina et al. discuss current approaches to assess neurogenesis and prospects in the application of optogenetic protocols to restore neurogenesis in patients with Parkinson’s disease [59].

In their paper, Ivashkina et al. present a methodological development and experimental application of a genetically engineered optosensoric tool that allows the imaging of activity-induced neuronal c-fos expression [60]. The authors show a novel light-controlled approach for the long-term analysis of calcium activity in the cortical neurons that were specifically tagged through c-fos expression during a particular cognitive episode. In addition, using in vivo two-photon imaging of Fos-GFP PtA neurons, they report specific changes in neuronal activity in this cortical area during both the acquisition and retrieval of associative fear memory. Taken together, these results suggest that Fos-Cre-GCaMP mice are suitable for the investigation of calcium activity in the neurons which were specifically activated during a particular learning episode.

In the work by Zhilyakov et al., the authors reported the results from their investigation on the relationship between the nicotine-induced autoregulation of acetylcholine (ACh) release and the changes in the concentration of presynaptic calcium levels [61]. For this purpose, they used a pharmacological approach, electrophysiological techniques, and a method for the optical registration of changes in calcium levels in the motor nerve endings. The authors found that an agonist of nicotinic receptors (at a concentration not significantly affecting the state of the postsynaptic membrane) leads to a decrease in the amount of released ACh quanta. This effect is accompanied not by a decrease but by an increase in calcium ion entry into the motor nerve terminal. These results suggest that the nicotinic cholinergic receptors responsible for the mechanism of ACh release autoregulation are the receptors of neuronal type. Activation of these receptors leads to the upregulation of the Cav1 type of VGCCs, resulting in the enhancement of Ca^2+^ entry into the nerve ending. This study adds new elements to the understanding of the cholinergic system functioning and the envisioning of novel potential approaches for the treatment of diseases associated with cholinergic dysfunctions.

In the field of optosensorics, Ponomareva et al. described the effectiveness of the genetically encoded biosensor, ClopHensor, expressed in transgenic mice for the estimation of [H^+^]_i_ and [Cl^−^]_i_ concentrations in brain slices [62]. They performed simultaneous monitoring of [H^+^]_i_ and [Cl^−^]_i_ under different experimental conditions, including changing external concentrations of ions (Ca^2+^, Cl^−^, K^+^, Na^+^) and the synaptic stimulation of Shaffer’s collaterals of hippocampal slices. The results obtained illuminate the different pathways regulating Cl^−^ and pH equilibrium in neurons and demonstrate that ClopHensor, expressed in transgenic mice, represents an efficient tool for the non-invasive monitoring of intracellular Cl^−^ and H^+^ ions.

In their work, Sotskov et al. performed in vivo imaging of neuronal activity in the CA1 field of the mouse hippocampus using genetically encoded green calcium indicators, including the novel NCaMP7 and FGCaMP7, designed specifically for in vivo calcium imaging [63]. The purpose of this study was to investigate the dynamics of the initial place field formation in the mouse hippocampus. Their data show that neuronal activity recorded with genetically encoded calcium sensors revealed fast behaviour-dependent plasticity in the mouse hippocampus, resulting in the rapid formation of place fields and population activity that allowed the reconstruction of the geometry of the navigated maze. Taken together, these results reveal the fast emergence and tuning dynamics of place cell codes and demonstrate the applicability of novel calcium indicators NCaMP7 and FGCaMP7 in the light-controlled analysis of neural functions in behaving mice.

In the field of photopharmacology, Matera et al. reported a novel photoswitchable ligand that enables reversible spatiotemporal control of dopaminergic transmission [64]. They demonstrated that this new photoswitch, named azodopa, activates D_1_-like receptors in vitro in a light-dependent manner. Moreover, azodopa enables reversibly photocontrolling zebrafish motility on a timescale of seconds and allows separating the retinal component of dopaminergic neurotransmission. Finally, they proved that azodopa increases the overall neural activity in the cortex of anesthetized mice and displays illumination-dependent activity in individual neurons. Azodopa is the first photoswitchable dopamine agonist with demonstrated efficacy in wild-type animals and opens the way forward to remotely controlling dopaminergic neurotransmission for investigational and therapeutic purposes.

Finally, the review article by Nin-Hill et al. discusses the application of structure-based computational methods, such as homology modelling, molecular docking, molecular dynamics, and enhanced sampling techniques, to photoswitchable ligands targeting voltage- and ligand-gated ion channels [65]. Notably, the examples presented by the authors show how the integration of computational modelling with experimental data can greatly facilitate photoswitchable ligand design and optimization and provide structural insights to understand the observed light-regulated effects. They conclude by stating that the latest advances in structural biology will further support computer-assisted approaches in photopharmacology.

Overall, this Special Issue of the *International Journal of Molecular Sciences* contains original contributions reporting recent advances in the fields of optogenetics, optosensorics, and photopharmacology, as well as review papers discussing key achievements and prospects in the field. Therefore, we expect that these articles will be of interest to many scientists working with light-based biological methods and will inspire further investigations in relevant research areas.
